# Is There a Female Protective Effect Against Attention-Deficit/Hyperactivity Disorder? Evidence From Two Representative Twin Samples

**DOI:** 10.1016/j.jaac.2016.04.004

**Published:** 2016-06

**Authors:** Mark J. Taylor, Paul Lichtenstein, Henrik Larsson, Henrik Anckarsäter, Corina U. Greven, Angelica Ronald

**Affiliations:** a Karolinska Institutet, Stockholm; b Centre for Ethics, Law and Mental Health (CELAM), University of Gothenburg, Gothenburg, Sweden; c Radboud University Medical Center, Donders Institute for Brain, Cognition and Behaviour, Nijmegen, The Netherlands; Karakter Child and Adolescent Psychiatry Center, Nijmegen; and King’s College London, Medical Research Council Social, Genetic and Developmental Psychiatry Centre, Institute of Psychiatry, Psychology and Neuroscience, London; d Centre for Brain and Cognitive Development, Birkbeck, University of London

**Keywords:** ADHD, sex differences, genetics, twin study

## Abstract

**Objective:**

Attention-deficit/hyperactivity disorder (ADHD) is more frequent in males than in females. The “female protective effect” posits that females undergo greater exposure to etiological factors than males in order to develop ADHD, leading to the prediction that relatives of females with ADHD will display more ADHD behaviors. We thus tested whether cotwins of females displaying extreme ADHD traits would display more ADHD traits than cotwins of males displaying extreme ADHD traits.

**Method:**

Parents of approximately 7,000 pairs of nonidentical twins in Sweden, and approximately 4,000 pairs of twins in England and Wales, completed dimensional assessments of ADHD traits. Probands were selected on the basis of scoring within the highest 10% of the distribution in each sample. Dimensional scores of cotwins of probands, as well as the categorical recurrence rate, were investigated by proband sex.

**Results:**

Cotwins of female probands displayed higher mean ADHD trait scores (mean = 0.62−0.79) than cotwins of male probands (mean = 0.38−0.55) in both samples. This trend was significant in the Swedish sample (*p* < .01) and when the 2 samples were merged into a single, larger sample (*p* < .001). When the samples were merged, there was also a significant association between proband sex and cotwin’s categorical status, with more cotwins of female probands also being probands than cotwins of male probands.

**Conclusion:**

These findings support a female protective effect against ADHD behaviors, suggesting that females require greater exposure to genetic and environmental factors associated with ADHD in order to develop the condition.

Attention-deficit/hyperactivity disorder (ADHD) is a neurodevelopmental condition characterized by excessive hyperactivity and impulsivity, inattentiveness, or a combination of these symptoms.^1^ Epidemiological studies suggest that, overall, ADHD affects between 5% and 7% of the population.^2^ Notably, ADHD appears to be substantially more common in males than in females. A study of 10 European countries, for instance, indicated that males with ADHD outnumbered females with ADHD by ratios from 2 to 1 to 16 to 1.^3^ The excess of males with ADHD has been further confirmed by meta-analyses, with 4 times as many males as females thought to be affected.^2^, ^4^

A number of twin studies have established that ADHD is among the most heritable of neuropsychiatric conditions.^5^, ^6^, ^7^, ^8^, ^9^ The high heritability of ADHD does not vary markedly whether it is conceptualized as a categorical, diagnosed condition^5^, ^6^ or treated as a continuous trait in the general population,^7^, ^9^ thus indicating that severe forms of ADHD may be linked genetically with milder, subclinical traits of ADHD in the general population. Such studies, however, have yet to shed light on the reasons why ADHD appears to be so much more common in males than in females.

One possible explanation for the sharp sex discrepancy in ADHD prevalence is a putative “female protective effect” model. Under this model, females would be predicted to require greater exposure than males to genetic and environmental factors associated with ADHD in order to display sufficient ADHD behaviors to warrant a diagnosis, thus meaning that fewer females than males would be expected to be diagnosed with ADHD.^10^, ^11^ As such, one would expect more causal factors to be present in the families of females with ADHD, leading to the prediction that ADHD and ADHD behaviors would be more prevalent in the relatives of females with ADHD. The female protective effect is presently receiving considerable attention in relation to autism spectrum disorders (ASD), which are similarly male-biased conditions. In 1 study, for example, the fraternal cotwins of females displaying a high degree of autistic traits displayed more autistic traits than did cotwins of males with high degrees of autistic traits, and were also more likely to display high scores themselves.^12^

Very few studies have tested for the existence of a female protective effect against ADHD. A recent Swedish investigation suggested that merely having a female cotwin is associated with displaying a greater degree of ADHD traits than having a male cotwin, although this study did not take account of the degree to which the index twin displayed ADHD symptoms.^13^ In 1 study of a US-based twin sample, traits of ADHD were examined in the cotwins of individuals displaying a high degree of traits of ADHD. Cotwins of females displaying extreme ADHD traits displayed significantly greater ADHD-like behaviors than cotwins of males displaying extreme traits of ADHD.^14^ Of note, however, the effect was not present for the cotwins of the most severely affected twins, perhaps owing to the small effect size and lower number of twins displaying the very highest scores.

As a consequence, we aimed to test for the existence of a female protective effect against ADHD behaviors in 2 independent, large-scale European twin samples. We first tested whether the cotwins of females displaying extreme degrees of ADHD traits would exhibit more continuous ADHD traits than the cotwins of high-scoring males. Second, we sought to test whether high-scoring female twins would be more likely to have a high-scoring cotwin than high-scoring male twins. The 1 previous study documenting this effect reported that the effect size was small^14^; thus, we not only aimed to test for the female protective effect against ADHD in our 2 samples independently, but also pooled the 2 samples to increase power. We expected, in light of existing evidence, to find evidence of a female protective effect against ADHD behaviors.^13^, ^14^

## Method

### Participants

Data were collected from participants in 2 representative, community-based twin studies. The Child and Adolescent Twin Study in Sweden (CATSS) is a study of twins born in Sweden since 1992. Initially, the twins were contacted in connection with their ninth birthdays.^15^ For the present study, data were collected from twins participating in CATSS when they were aged 9 years. The second sample comprised participants in the Twins Early Development Study (TEDS); TEDS is a sample of twins born in England and Wales between 1994 and 1996.^16^ Data for the present study were collected from TEDS participants when twins were aged 8 years. TEDS and CATSS are representative of the populations of England and Wales and of Sweden, respectively.^15^, ^16^

Both CATSS and TEDS comprise both monozygotic (MZ) and dizygotic (DZ) twins, although only DZ twins were included in this study owing to the fact that the genetic resemblance of 2 DZ twins within a pair is the same as the resemblance between 2 singleton siblings (approximately 50% of their segregating DNA code on average). Both same-sex and opposite-sex DZ twins were included. In CATSS, families of 6,817 pairs of DZ twins returned data, whereas 4,309 participating families in TEDS returned data. In CATSS, exclusions were conducted for known brain injuries and chromosomal syndromes (n = 113), leaving 6,704 DZ twin pairs. Participants in TEDS were excluded for genetic and chromosomal syndromes, extreme perinatal complications, and missing first contact data (n = 254), leaving 4,055 pairs of DZ twins. Combined, there were 10,759 DZ twin pairs across the 2 samples.

CATSS has ethical approval from the Karolinska Institutet Ethical Review Board, and TEDS has ethical approval from the King’s College London, Institute of Psychiatry, Psychology and Neuroscience Ethics Committee.

### Measures

In CATSS, the ADHD modules of the Autism-Tics, ADHD, and Other Comorbidities inventory (A-TAC)^17^ were administered to parents of the twins over the telephone. There are 2 ADHD modules, assessing hyperactivity/impulsivity and inattentiveness, comprising a total of 19 items that correspond closely to *DSM-IV* criteria for ADHD.^1^ Each item comprised a question, answered “yes” (for a score of 1), “yes, to some extent” (for a score of 0.5), or “no” (for a score of 0). Thus, the maximum possible score was 19. In the sample of DZ twins used in the present study, the A-TAC ADHD module had strong internal consistency (α = 0.92). A prior study reported strong construct validity for the scale, with 92% sensitivity and 75% specificity for detecting ADHD.^18^ The A-TAC also comprises 2 subscales, assessing ADHD subtypes: Hyperactivity/Impulsivity (10 items) and Inattention (9 items).

Parents of twins participating in TEDS completed the ADHD subscale of the Conners’ Parent Rating Scale–Revised (Conners ADHD).^19^ The measure was mailed to parents of the twins, who completed and returned it. The Conners ADHD measure comprises 18 items that are closely linked with the *DSM-IV* criteria for ADHD.^1^ Each item comprised a statement in response to which the parents rated, on a 0 to 3 scale, the extent to which each item was true of their children. The maximum possible score was 54. In the present study, the Conners ADHD showed strong internal consistency (α = 0.91). Previously, individuals with ADHD have been shown to score more highly on the measure than controls,^19^ supporting its construct validity. As with the A-TAC, Conners ADHD comprises Hyperactivity/Impulsivity and Inattention subscales (9 items each).

### Data Analysis

#### Proband Selection

In both samples, 1 twin was randomly selected as the “index twin.” All other twins were cotwins. Probands were selected as the index twins scoring within the highest 10% of the A-TAC and Conners ADHD distributions, with such a cut-off designed to maximize statistical power while capturing severe-enough cases. Thus, probands in CATSS were selected on the basis of A-TAC scores of 6.5 or more, whereas TEDS probands were defined as index twins scoring at least 23 on the Conners ADHD. Subsequently, analyses were repeated using more conservative cut-offs of 9.5 on the A-TAC and 28 on the Conners ADHD. These cut-offs were designed to capture the highest scoring 5% of each sample, thus testing for a female protective effect in relation to even more extreme scores. Due to the lack of sex-specific diagnostic criteria for ADHD, the same cut-offs were used to select probands, regardless of sex. The number of probands, split by sex, is given in Table 1.

#### Statistical Analysis

To test whether cotwins of female probands would display higher ADHD trait scores than cotwins of male probands, 3×2 between-subjects analysis of variance (ANOVA) was used. Proband status of the index twin (male proband, female proband, or control) was the grouping variable, with cotwins’ ADHD trait scores acting as the outcome variable. An omnibus test initially compared scores across cotwins of male probands, female probands, and controls, before planned comparisons compared the scores of cotwins of male and female probands. Individual *p* values were adjusted for multiple comparisons within each sample using the Bonferroni correction. All reported *p* values are adjusted in this manner. Effect sizes were summarized using Cohen’s *d.* Effect sizes were interpreted using Cohen’s criteria,^20^ with *d* of 0.20 to 0.49 considered a small effect, 0.50 to 0.79 medium, and greater than 0.80 large.

To test whether the sex of the probands was associated with whether or not their cotwin would also be a proband, categorical analyses were used. Using the above identified cut-offs, cotwins were classified as either “affected” (i.e., scoring above a given cut-off) or “unaffected” (i.e., scoring below a given cut-off). Then χ^2^ tests of association were used to test whether cotwin status was significantly associated with proband sex. Effect sizes were summarized using odds ratios (ORs).

Analyses were first conducted separately in CATSS and TEDS. To bolster statistical power, a third set of analyses was performed on the 2 samples combined. The Conners ADHD and A-TAC were both heavily, positively skewed and were therefore log transformed before analysis (Table 2). All cotwin scores used in analyses were standardized by sex of the cotwin, thus ensuring that cotwin sex was controlled for and allowing easier comparability of findings across samples. All analyses were performed in R.^21^

Post hoc analyses subsequently tested for a female protective effect against specific ADHD behaviors. All of the analyses detailed above were repeated on the Hyperactivity/Impulsivity and Inattention subscales of the A-TAC and Conners ADHD. Because these analyses were post hoc, *p* values were not adjusted for multiple comparisons.

## Results

Descriptive statistics for the A-TAC and Conners ADHD are given in Table 2.

### Analysis of Continuous Scores

**Figure 1:**
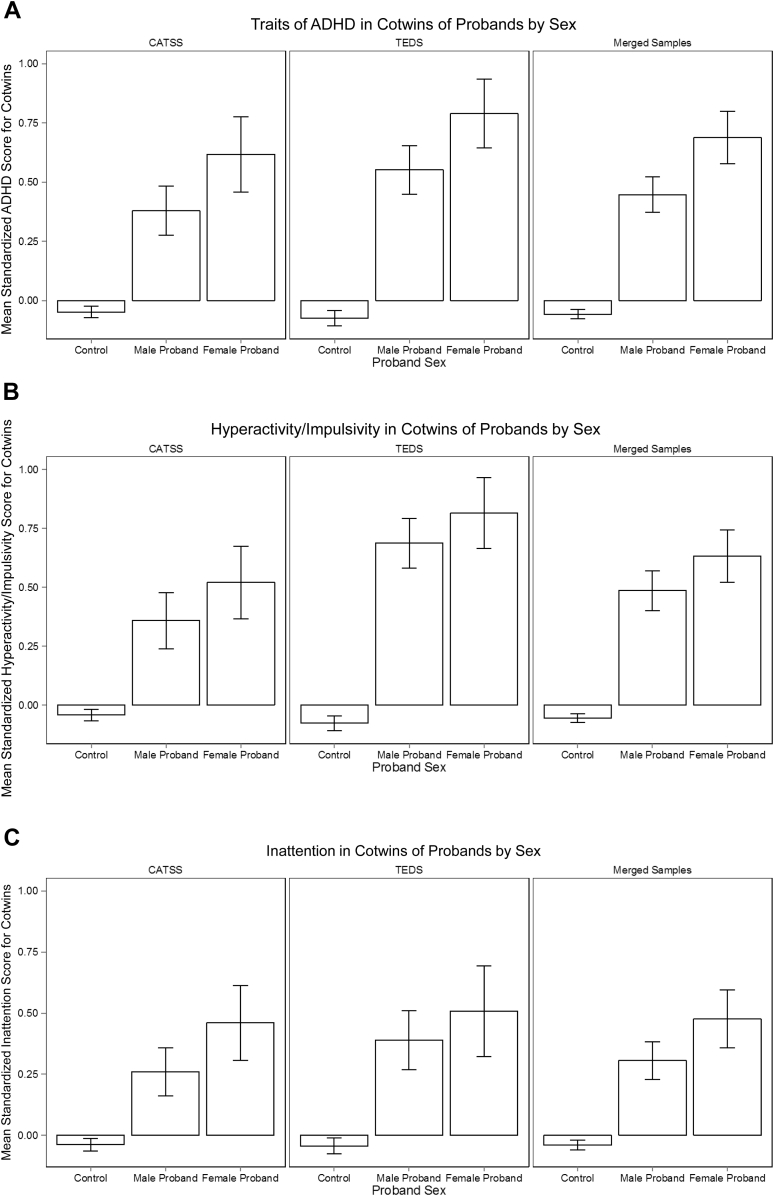
Mean cotwin scores for (a) full-scale attention-deficit/hyperactivity disorder (ADHD), (b) hyperactivity/impulsivity, and (c) inattention for the highest scoring 10% in all analyses. Note: Error bars represent standard deviations. CATSS = Child and Adolescent Twin Study in Sweden; TEDS = Twins Early Development Study.

Mean standardized scores of cotwins of male probands, female probands, and controls are all shown in Figure 1 for the analyses using the 10% cut-offs. In CATSS, scores differed significantly across the 3 groups (*F*_2,6688_ = 79.35, *p* < .01), with cotwins of female probands scoring highest (mean = 0.62), followed by cotwins of male probands (mean = 0.38), and cotwins of controls (mean = −0.05). Specifically, cotwins of female probands displayed significantly higher A-TAC scores than cotwins of male probands (*t*_6688_ = −2.84, *p* < .01), with a modest effect size of *d* = 0.07.

Similarly in TEDS, Conners ADHD scores differed significantly across cotwins of male probands (mean = 0.55), cotwins of female probands (mean = 0.79), and cotwins of controls (mean = −0.07) (*F*_2,4040_ = 102.30, *p* < .001). Planned contrasts, however, indicated that mean Conners ADHD scores were not significantly elevated in cotwins of female probands relative to cotwins of male probands (*t*_4040_ = −2.36, *p* = .08, *d* = 0.07), despite a trend in this direction.

Merging the 2 samples produced the same pattern; mean ADHD trait scores differed significantly in the 3 groups (*F*_2,10731_ = 175.90, *p* < .001), with cotwins of female probands showing the highest ADHD trait scores (mean = 0.69), followed by cotwins of male probands (mean = 0.45), and controls (mean = −0.06). The planned contrast confirmed that mean ADHD trait scores were significantly higher for cotwins of female probands than cotwins of male probands (*t*_10731_ = −3.73, *p* < .001, *d* = 0.07).

All mean ADHD trait scores for cotwins of probands selected under the more severe, 5% cut-offs are given in Table 3. Merging the 2 samples produced the same pattern of results as the 10% cut-off. Index twin status exacted a significant main effect on the mean ADHD trait scores of cotwins (*F*_2,10731_ = 95.90, *p* < .001), with cotwins of female probands displaying the highest ADHD trait scores (mean = 0.73), followed by cotwins of male probands (mean 0.51) and controls (mean = −0.03). Mean ADHD trait scores were significantly elevated in cotwins of female probands compared with cotwins of male probands (*t*_10731_ = −2.38, *p* < .05, *d* = 0.05).

Using a 5% cut-off, the same pattern emerged in each individual sample. In CATSS, mean A-TAC scores differed significantly across cotwins of male probands (mean = 0.43), female probands (mean = 0.69), and controls (mean = −0.03) (*F*_2,6688_ = 46.39, *p* < .01); however, mean A-TAC scores for cotwins of female probands were not significantly higher than mean A-TAC scores for cotwins of male probands (*t*_6688_ = −2.10, *p* = .16, *d* = 0.05). The same result emerged for TEDS. Although the main effect of index twin status was significant (*F*_2,4040_ = 51.63, *p* < .001), with cotwins of female probands showing the highest mean Conners ADHD scores (mean = 0.79), followed by cotwins of male probands (mean = 0.62) and controls (−0.04), mean Conners ADHD scores for cotwins of female probands were not significantly higher than mean scores for cotwins of male probands (*t*_4040_ = −1.10, *p* = .27, *d* = 0.03).

### Analysis of Categorical Recurrence

Table 4 shows the number of affected and unaffected cotwins by proband sex for each sample and cut-off. Using a 10% cut-off to select probands, the association between proband sex and cotwin status was significant only when CATSS and TEDS were merged (χ^2^_1_ = 5.21, *p* < .05, OR = 0.70 [95% CI: 0.54/0.94]), with a greater proportion of cotwins of female probands (29%) than cotwins of male probands (22%) showing higher ADHD trait scores.

In CATSS, a greater proportion of cotwins of female probands scoring above the 10% also scored above the cut-off (15% of cotwins of female probands compared with 9% of cotwins of male probands). The association was small and nonsignificant, however (χ^2^_1_ = 4.67, *p* = .06, OR = 0.57 [95% CI: 0.34/0.92]). Similarly in TEDS, 38% of cotwins of female probands scored above the 10% cut-off compared with 29% of cotwins of male probands, although this association was again small and failed to reach significance (χ^2^_1_ = 3.23, *p* = .14, OR = 0.66 [95% CI: 0.43/1.01]).

The findings followed the same pattern when a cut-off that selected 5% of index twins as probands was used. When CATSS and TEDS were merged, there was a significant association between proband sex and cotwin status (χ^2^_1_ = 6.38, *p* ≤ .05, OR = 0.54 [95% CI: 0.35/0.86]), with a greater proportion of cotwins of female probands (26%) than cotwins of male probands (16%) showing a pronounced degree of ADHD traits. In CATSS alone, more cotwins of female probands (21%) than cotwins of male probands (12%) were affected, yet this association was not significant (χ^2^_1_ = 3.35, *p* = .14, OR = 0.53 [95% CI: 0.28/0.99]). The same was true of TEDS: more cotwins of female probands (33%) than cotwins of male probands (22%) were affected, yet this seeming association was not significant (χ^2^_1_ = 2.46, *p* = .24, OR = 0.56 [95% CI: 0.29/1.08]).

### Subscale Analyses

Figures 1b and 1c show the mean scores of cotwins of probands on the A-TAC and Conners ADHD subscales (Hyperactivity/Impulsivity and Inattention), with probands scoring within the highest 10% of the subscales. In CATSS, TEDS, and the combined cohorts, cotwins of female probands displayed the highest mean Hyperactivity/Impulsivity scores, followed by cotwins of male probands and cotwins of controls. In all analyses, the mean scores of cotwins of female probands were significantly higher than those of the other 2 groups (*p* < .05).

Inattention followed the same pattern, as shown in Figure 1c. Cotwins of female probands displayed the highest mean Inattention score, followed by cotwins of male probands and cotwins of controls. The mean scores of cotwins of female probands were significantly higher than both other groups of cotwins in all 3 analyses (*p* < .05).

These results are shown in full in Table S1, Table S2, Table S3, Table S4, available online.

## Discussion

This investigation sought to test whether a female protective effect can account for the substantially elevated prevalence of ADHD in males relative to females.^2^, ^3^, ^4^ The results of this study lend partial credence to a female protective effect hypothesis for ADHD. In line with the results of an existing US study^14^ and our hypotheses, there was some evidence to indicate that the cotwins of females displaying an extreme degree of characteristic ADHD behaviors displayed more such behaviors themselves than did the cotwins of males showing an extreme degree of ADHD traits. Furthermore, cotwins of females with particularly high ADHD trait scores were more likely to display an extreme degree of ADHD behaviors than were the cotwins of males. As such, these findings tentatively indicate that a female protective effect could be a potentially viable model to help understand the development of ADHD.

Our findings provide a platform for future research into the genetic basis of ADHD to build upon. Although twin studies of ADHD have consistently supported its high heritability,^5^, ^6^, ^7^, ^8^, ^9^ elucidating the precise genetic mechanisms underpinning ADHD has proved elusive.^22^ The female protective effect model provides an opportunity to raise further research questions in such research. For example, genes can be divided into high-impact and low-impact sets.^23^ One possibility is that females with ADHD are more likely to inherit higher impact genes associated with ADHD, which are rarer. To illustrate, ASD also more commonly affects males than females,^24^ and recent twin and family studies support a female protective effect against ASD.^12^, ^25^ A genetic study then indicated that females with ASD displayed a higher degree of larger copy number variants, which were more likely to be maternally inherited.^11^ Similar studies of ADHD may well prove useful in furthering our understanding of the etiology of ADHD.

Indeed, although our study did not investigate any specific etiological mechanisms associated with ADHD, our findings suggest that investigating the degree of exposure to etiological factors associated with ADHD in males and females with the condition may be a worthwhile future research direction. Although the above example mentioned larger, rarer copy number variants, one might also test whether females with ADHD exhibit a greater number of smaller, common genetic variants. Indeed, in using polygenic scores, which have yielded useful insights in the genetic architecture of ADHD,^26^ one could investigate whether females with ADHD display a greater degree of common genetic variants associated with ADHD than males with ADHD.^27^

One could also extend this to causal environmental factors. Although twin studies indicate that genetic factors seem to outweigh environmental factors in the etiology of ADHD,^5^, ^6^, ^7^, ^8^, ^9^ research has implicated certain environmental exposures with ADHD. For instance, lower birth weight is thought to be a causal environmental factor in ADHD.^28^, ^29^ It may be that females with ADHD undergo greater exposure to such factors compared with males; for instance, could females with ADHD display an even lower birth weight than males with ADHD?

The presence of a female protective effect against ADHD behaviors also has implications for clinical practice. If clinicians take account of family history when diagnosing ADHD, it may be beneficial also to take into account the sex of any previously affected relatives, under the assumption that relatives of females with ADHD are more likely to exhibit ADHD symptoms than relatives of males with ADHD. The caveat to this assertion, however, is that our findings are based only on twin data. The female protective effect against ADHD needs to be replicated in alternative, non-twin samples before such a conclusion can be decisively drawn. For instance, a recent study of ASD found that siblings of female non-twins with ASD were more likely to have ASD than siblings of male non-twins.^25^ Such studies of non-twin relatives are now needed in relation to ADHD.

It does need to be noted that the overall sizes of the effects reported here, where significant, were weak. The effect reported in this article is less than half the size of the female protective effect in relation to autism reported in a similar study.^12^ Indeed, significant findings emerged only for the more severe cut-off of 5% when the 2 samples used were merged to create a larger sample. The small effect size seen here is consistent with that reported previously,^14^ and so it is quite clear that subsequent studies testing the female protective effect model of ADHD are going to require large samples.

The small female protective effect seen here does, nevertheless, stress the need not to discount alternative explanations for the increased number of males with ADHD relative to females. There is very limited research considering phenotypic differences between males and females with ADHD. For instance, 1 study investigated sex differences in ADHD across 10 European countries, and reported that females with ADHD displayed more emotional difficulties.^3^ Furthermore, the *DSM-IV* criteria for ADHD, upon which our measures were based, are based exclusively on observations of males.^1^, ^30^, ^31^

Two further possibilities cannot be discounted from our study. Although our findings lend support to the notion of a female protective effect against ADHD, it is not mutually exclusive to the hypothesis that males have more risk factors for ADHD. It is also, in theory, possible that rater contrast effects drove the higher scores seen in cotwins of female probands. Rater contrast effects refer to the scenario whereby parent ratings of 1 twin are influenced by how they view their cotwin.^32^ To create the observed pattern of results, parents would need to have shown a stronger rater contrast effect on the cotwins (who were both male and female) of male probands than of female probands. In twin analyses of the A-TAC and Conners ADHD scale, rater contrast effects have been modeled and shown to be modest,^9^, ^33^ suggesting that rater contrast effects are unlikely to be an adequate explanation for our findings.

In addition to the caveat regarding the small effect size, our study did have further limitations that need taking into account. Proband status was ascertained through the use of dimensional questionnaire measures, as opposed to in-depth assessments of ADHD. The use of this approach would, however, have come at the cost of the large sample size. As mentioned above, only twins were used in this study. Although we removed MZ twins to ensure that the genetic relatedness of the relatives in our sample was similar to that of fraternal siblings, it is important to know whether these findings extend to non-twin relatives in the future. In defense of our use of a twin sample, on the other hand, there is evidence to indicate that ADHD traits are not elevated in twins’ relatives to singletons.^34^

Finally, our study did not take comorbidity into account. Females with ADHD are more likely to present with additional disorders, such as anxiety and depression, than males with ADHD.^35^ If one assumes a certain degree of common causal factors across ADHD and other neuropsychiatric disorders, as supported by recent twin studies,^36^, ^37^ then it is possible that females manifest with different symptoms at lower levels of exposure to etiological factors, with ADHD emerging only after greater exposure. This could be tested in the future using ADHD polygenic risk scores, for example.

To a certain degree, this study indicates that females are protected against behaviors characteristic of ADHD. Although our findings do not speak to any specific mechanisms through which this effect may operate, this study indicates that further research on the female protective effect model is warranted in relation to ADHD, with a view to identify the specific biological basis of this effect. If the effect holds across multiple epidemiological methods, then it represents a plausible explanation for why fewer females than males develop ADHD, as well as assisting in the diagnostic process.

## Figures and Tables

**Table S1:** Analyses of Hyperactivity/Impulsivity Continuous Scores

	Continuous Scores (5%)		
CATSS	TEDS	Merged Samples
Cotwin of male proband mean	0.36 (1.19)	0.74 (0.85)	0.51 (1.08)
Cotwin of female proband mean	0.72 (1.23)	0.99 (0.85)	0.83 (1.10)
Cotwin of control mean	−0.02 (0.98)	−0.04 (0.99)	−0.03 (0.98)
Omnibus ANOVA	*F*_2,6696_ = 41.65, *p* < .001	*F*_2,4038_ = 77.06, *p* < .001	*F*_2,10737_ = 109.60, *p* < .001
Planned contrast	*t*_6696_ = −3.04, *p* < .01, *d* = 0.04	*t*_4038_ = −1.76, *p* = .08, *d* = 0.05	*t*_10737_ = −10.06, *p* < .001, *d* = 0.19

	Continuous Scores (10%)		
CATSS	TEDS	Merged Samples
Cotwin of male proband mean	0.36 (1.18)	0.69 (0.84)	0.49 (1.07)
Cotwin of female proband mean	0.52 (1.18)	0.81 (0.90)	0.63 (1.09)
Cotwin of control mean	−0.04 (0.97)	−0.08 (0.98)	−0.06 (0.97)
Omnibus ANOVA	*F*_2,6696_ = 62.08, *p* < .001	*F*_2,4038_ = 121.20, *p* < .001	*F*_2,10737_ = 167.30, *p* < .001
Planned contrast	*t*_6696_ = −7.66, *p* < .001, *d* = 0.09	*t*_4038_ = −11.85, *p* < .001, *d* = 0.37	*t*_10737_ = −13.31, *p* < .001, *d* = 0.26

Note: Numbers in parentheses are standard deviations. ANOVA = analysis of variance; CATSS = Child and Adolescent Twin Study in Sweden; TEDS = Twins Early Development Study.

**Table S2:** Analyses of Hyperactivity/Impulsivity Categorical Recurrence Rates

CATSS	5%		10%	
“Affected” Cotwin	“Unaffected” Cotwin	“Affected” Cotwin	“Unaffected” Cotwin
Male proband	23 (11)	185 (89)	41 (11)	341 (89)
Female proband	18 (18)	82 (82)	30 (13)	199 (87)
	*χ*^2^_1_ = 2.25, *p* = .13, OR = 0.57 [0.29−1.11]		*χ*^2^_1_ = 0.57, *p* = .45, OR = 0.80 [0.48−1.31]	

TEDS	5%		10%	
“Affected” Cotwin	“Unaffected” Cotwin	“Affected” Cotwin	“Unaffected” Cotwin
Male proband	30 (21)	112 (79)	62 (25)	180 (75)
Female proband	19 (29)	46 (71)	50 (36)	90 (64)
	*χ*^2^_1_ = 1.20, *p* = .27, OR = 0.64 [0.33−1.27]		*χ*^2^_1_ = 3.89, *p* = .05, OR = 0.62 [0.40−0.97]	

Merged Samples	5%		10%	
“Affected” Cotwin	“Unaffected” Cotwin	“Affected” Cotwin	“Unaffected” Cotwin
Male proband	53 (15)	297 (85)	131 (21)	493 (79)
Female proband	37 (22)	128 (78)	101 (27)	268 (73)
	*χ*^2^_1_ = 3.63, *p* = .06, OR = 0.68 [0.39−0.99]		*χ*^2^_1_ = 4.92, *p* < .05, OR = 0.71 [0.52−0.95]	

Note: Data are shown as n (%) except where noted and in brackets, which are 95% CIs. CATSS = Child and Adolescent Twin Study in Sweden; OR = odds ratio; TEDS = Twins Early Development Study.

**Table S3:** Analyses of Inattention Continuous Scores

	5%		
CATSS	TEDS	Merged Samples
Cotwin of male proband mean	0.30 (1.09)	0.31 (1.01)	0.30 (1.06)
Cotwin of female proband mean	0.57 (1.28)	0.44 (1.15)	0.53 (1.24)
Cotwin of control mean	−0.02 (0.99)	−0.02 (0.99)	−0.02 (0.99)
Omnibus ANOVA	*F*_2,6691_ = 29.71, *p* < .001	*F*_2,4038_ = 13.17, *p* < .001	*F*_2,10732_ = 42.56, *p* < .001
Planned contrast	*t*_6691_ = −2.37, *p* < .05, *d* = 0.03	*t*_4038_ = −0.84, *p* = .40, *d* = 0.03	*t*_10732_ = −6.18, *p* < .001, *d* = 0.12

	10%		
CATSS	TEDS	Merged Samples
Cotwin of male proband mean	0.26 (1.09)	0.39 (0.97)	0.31 (1.05)
Cotwin of female proband mean	0.46 (1.20)	0.51 (1.00)	0.48 (1.13)
Cotwin of control mean	−0.04 (0.98)	−0.04 (0.99)	−0.04 (0.98)
Omnibus ANOVA	*F*_2,6691_ = 46.07, *p* < .001	*F*_2,4038_ = 38.50, *p* < .001	*F*_2,10732_ = 83.02, *p* < .001
Planned contrast	*t*_6691_ = −6.28, *p* < .001, *d* = 0.15	*t*_4038_ = −6.75, *p* < .001, *d* = 0.21	*t*_10732_ = −9.06, *p* < .001, *d* = 0.17

Note: Numbers in parentheses are standard deviations. ANOVA = analysis of variance; CATSS = Child and Adolescent Twin Study in Sweden; TEDS = Twins Early Development Study.

**Table S4:** Analyses of Inattention Categorical Recurrence Rates

CATSS	5%		10%	
“Affected” Cotwin	“Unaffected” Cotwin	“Affected” Cotwin	“Unaffected” Cotwin
Male proband	22 (9)	216 (91)	80 (17)	388 (83)
Female proband	22 (20)	87 (80)	56 (26)	181 (74)
	*χ*^2^_1_ = 7.12, *p* < .05, OR = 0.40 [0.21−0.76]		*χ*^2^_1_ = 3.91, *p* = .05, OR = 0.67 [0.45−0.98]	

TEDS	5%		10%	
“Affected” Cotwin	“Unaffected” Cotwin	“Affected” Cotwin	“Unaffected” Cotwin
Male proband	14 (10)	127 (90)	45 (18)	210 (82)
Female proband	13 (23)	44 (77)	30 (26)	86 (74)
	*χ*^2^_1_ = 4.67, *p* < .05, OR = 0.37 [0.16−0.85]		*χ*^2^_1_ = 2.85, *p* = .09, OR = 0.61 [0.36−1.04]	

Merged Samples	5%		10%	
“Affected” Cotwin	“Unaffected” Cotwin	“Affected” Cotwin	“Unaffected” Cotwin
Male proband	36 (9)	343 (91)	125 (17)	598 (83)
Female proband	35 (21)	131 (79)	86 (32)	267 (68)
	*χ*^2^_1_ = 12.67, *p* < .001, OR = 0.39 [0.24−0.65]		*χ*^2^_1_ = 7.09, *p* < .05, OR = 0.65 [0.48−0.89]	

Note: Data are shown as n (%) except where noted and in brackets, which are 95% CIs. CATSS = Child and Adolescent Twin Study in Sweden; OR = odds ratio; TEDS = Twins Early Development Study.

**Table 1:** Numbers of Probands in Studies

Measure	Sample	5%		10%	
Male, n (%)	Female, n (%)	Male, n (%)	Female, n (%)
Total ADHD	CATSS	227 (70)	95 (30)	450 (69)	201 (31)
	TEDS	146 (70)	63 (30)	291 (68)	138 (32)
	Merged	373 (70)	158 (30)	741 (69)	339 (31)
Hyperactivity/Impulsivity	CATSS	208 (68)	100 (32)	382 (63)	229 (37)
	TEDS	142 (69)	65 (31)	242 (63)	140 (37)
	Merged	350 (68)	165 (32)	624 (63)	369 (37)
Inattention	CATSS	238 (69)	109 (31)	468 (66)	237 (34)
	TEDS	141 (71)	57 (29)	255 (69)	116 (31)
	Merged	379 (70)	166 (30)	723 (67)	353 (33)

Note: ADHD = attention-deficit/hyperactivity disorder; CATSS = Child and Adolescent Twin Study in Sweden; TEDS = Twins Early Development Study.

**Table 2:** Descriptive Statistics

Measure	Cronbach’s α	Possible Range of Scores	Mean Full Sample (SD)	Mean Males (SD)	Mean Females (SD)	Skew
A-TAC^a^	0.92	0−19	2.10 (3.21)	2.54 (3.54)	1.62 (2.73)	2.34
Conners ADHD^b^	0.91	0−54	10.84 (9.00)	12.67 (9.71)	9.04 (7.85)	1.37
A-TAC Hyp/Imp^c^	0.89	0−10	0.99 (1.69)	1.17 (1.85)	0.81 (1.48)	2.53
A-TAC Inatten^d^	0.90	0−9	1.03 (1.73)	1.28 (1.91)	0.78 (1.49)	2.23
Conners Hyp/Imp^e^	0.89	0−27	5.57 (4.93)	6.39 (5.28)	4.78 (4.41)	1.33
Conners Inatten^f^	0.91	0−27	5.27 (5.04)	6.29 (5.45)	4.26 (4.37)	1.41

Note: ADHD = attention-deficit/hyperactivity disorder; A-TAC = Autism-Tics and Other Comorbidities Inventory; Conners ADHD = ADHD subscale of the Conners’ Parent Rating Scale; Hyp = hyperactivity; Imp = impulsivity; Inatten = inattention.

a Mean A-TAC scores were significantly higher for males than females in the full sample (*t*_6539.22_ = 11.90, *p* < .001, *d* = 0.29).

b Mean Conners ADHD scores were significantly higher for males than females in the full sample (*t*_3843.64_ = 13.08, *p* < .001, *d* = 0.42).

c Mean A-TAC Hyperactivity/Impulsivity scores were significantly higher for males than females in the full sample (*t*_6431.70_ = 8.95, *p* < .001, *d* = 0.22).

d Mean A-TAC Inattention scores were significantly higher for males than females in the full sample (*t*_6369.05_ = 12.08, *p* < .001, *d* = 0.30).

e Mean Conners ADHD Hyperactivity/Impulsivity scores were significantly higher for males than females in the full sample (*t*_3890.60_ = 10.50, *d* = 0.34).

f Mean Connors ADHD Inattention scores were significantly higher for males than females in the full sample, t(_3829.86_) = 13.02, *p* < .001.

**Table 3:** Analysis of Continuous Traits of Attention-Deficit/Hyperactivity Disorder (ADHD) in Cotwins

	5%		
CATSS	TEDS	Merged Samples
Cotwin of male proband mean	0.43 (1.18)	0.62 (0.89)	0.51 (1.08)
Cotwin of female proband mean	0.69 (1.23)	0.79 (1.02)	0.73 (1.15)
Cotwin of control mean	−0.03 (0.98)	−0.04 (0.99)	−0.03 (0.99)
Omnibus ANOVA	*F*_2,6688_ = 46.39, *p* < .01	*F*_2,4040_ = 51.63, *p* < .001	*F*_2,10731_ = 95.90, *p* < .001
Planned contrast	*t*_6688_ = −2.10, *p* = .16, *d* = 0.05	*t*_4040_ = −1.10, *p* = .27, *d* = 0.03	*t*_10731_ = −2.38, *p* < .05, *d* = 0.05

	10%		
CATSS	TEDS	Merged Samples
Cotwin of male proband mean	0.38 (1.11)	0.55 (0.89)	0.45 (1.04)
Cotwin of female proband mean	0.62 (1.14)	0.79 (0.87)	0.69 (0.79)
Cotwin of control mean	−0.05 (0.97)	−0.07 (0.99)	−0.06 (0.98)
Omnibus ANOVA	*F*_2,6688_ = 79.35, *p* < .01	*F*_2,4040_ = 102.30, *p* < .001	*F*_2,10731_ = 175.90, *p* < .001
Planned contrast	*t*_6688_ = −2.84, *p* < .01, *d* = 0.07	*t*_4040_ = −2.36, *p* = .08, *d* = 0.07	*t*_10731_ = −3.73, *p* < .001, *d* = 0.07

Note: Merged samples are analyses of both Child and Adolescent Twin Study in Sweden (CATSS) and Twins Early Development Study (TEDS), merged into a single dataset. Numbers in parentheses are standard deviations. Omnibus analysis of variance (ANOVA) is a comparison of all 3 conditions (cotwins of male probands, cotwins of female probands, and cotwins of controls); planned contrast is a comparison of cotwins of male probands and cotwins of female probands.

**Table 4:** Analyses of Categorical Recurrence Rates

CATSS	5%		10%	
Cotwin “Affected”	Cotwin “Unaffected”	Cotwin “Affected”	Cotwin “Unaffected”
Male proband	28 (12)	199 (88)	40 (9)	410 (91)
Female proband	20 (21)	75 (79)	30 (15)	171 (85)
	*χ*^2^_1_ = 3.35, *p* = .14, OR = 0.53 [0.28/0.99]		*χ*^2^_1_ = 4.67, *p* = .06, OR = 0.57 [0.34/0.92]	

TEDS	5%		10%	
Cotwin “Affected”	Cotwin “Unaffected”	Cotwin “Affected”	Cotwin “Unaffected”
Male proband	32 (22)	114 (78)	83 (29)	208 (71)
Female proband	21 (33)	42 (67)	52 (38)	86 (62)
	*χ*^2^_1_ = 2.46, *p* = .24, OR = 0.56 [0.29/1.08]		*χ*^2^_1_ = 3.23, *p* = .14, OR = 0.66 [0.43/1.01]	

Merged Samples	5%		10%	
Cotwin “Affected”	Cotwin “Unaffected”	Cotwin “Affected”	Cotwin “Unaffected”
Male proband	60 (16)	313 (84)	163 (22)	578 (78)
Female proband	41 (26)	117 (74)	97 (29)	242 (71)
	*χ*^2^_1_ = 6.38, *p* < .05, OR = 0.54 [0.35/0.86]		*χ*^2^_1_ = 5.21, *p* < .05, OR = 0.70 [0.52/0.94]	

Note: Data are shown as n (%) except where noted and in brackets, which are 95% CIs. The percentages 5% and 10% indicate which cut-off was used to select probands in each analysis (highest scoring 10% of each sample or highest scoring 5% of each sample). Merged samples are analyses of both Child and Adolescent Twin Study in Sweden (CATSS) and Twins Early Development Study (TEDS) merged into a single dataset. OR = odds ratio.
